# Supplementary material for: Enhanced copper anticorrosion from Janus-doped bilayer graphene
Source: Nat Commun. 2023 Nov 17;14:7447. doi: 10.1038/s41467-023-43357-1 (PMC10656578; doi:10.1038/s41467-023-43357-1)
Supplement: Supplementary file 1 — Supplementary Information [file 41467_2023_43357_MOESM1_ESM.pdf]

Supplementary information for

Enhanced copper anticorrosion from Janus-doped bilayer graphene

Mengze Zhao, Zhibin Zhang, Wujun Shi, Yiwei Li, Chaowu Xue, Yuxiong Hu, Mingchao Ding, Zhiqun Zhang, Zhi Liu, Ying Fu, Can Liu, Muhong Wu, Zhongkai Liu, Xin-Zheng Li, Zhu-Jun Wang and Kaihui Liu

The supplementary information includes:

Supplementary Fig. 1-12

Supplementary Table 1

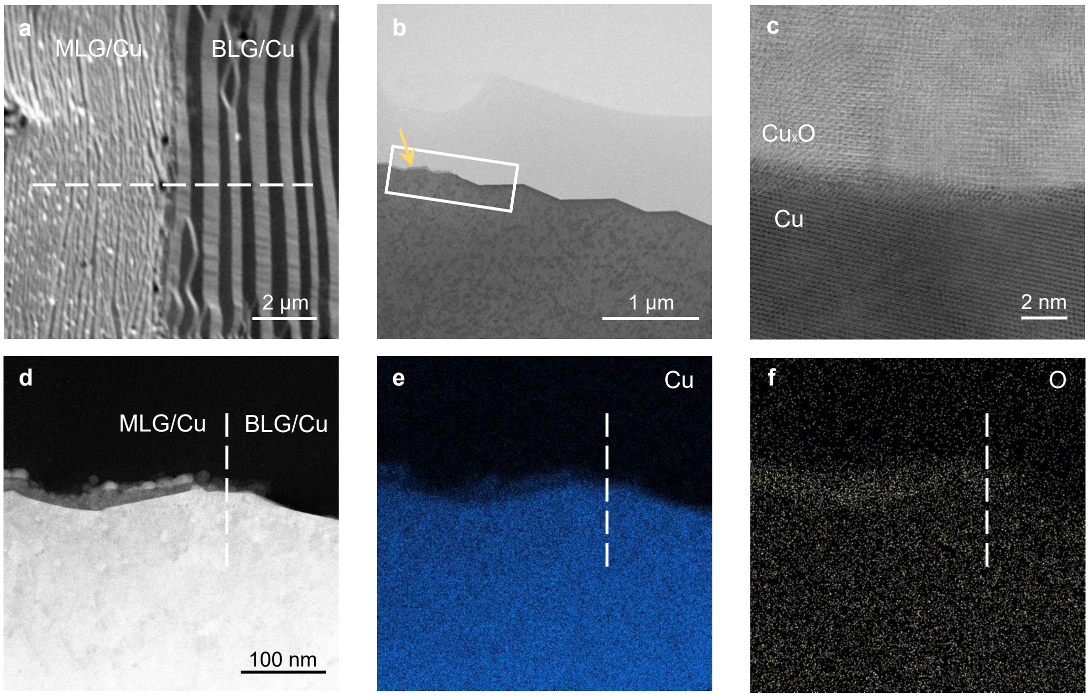

Supplementary Fig. 1 | Cross-sectional characterization at the boundary between monolayer and bilayer graphene coated Cu. a, Top-view SEM image showing the boundary between monolayer and bilayer graphene coated Cu. The dashed line indicates the cutting position of the focused ion beam experiment. **b**, Low-magnification cross-sectional STEM annular bright field (ABF) image of the monolayer-bilayer graphene coating boundary. The area framed by the rectangle corresponds to Fig. 1e. **c**, High-magnification STEM ABF image of the monolayer graphene coating region, corresponding to the yellow arrow pointed position in (b). **d-f**, TEM image around the boundary between monolayer and bilayer graphene-coated Cu and the corresponding EDS maps of Cu (e) and O (f).

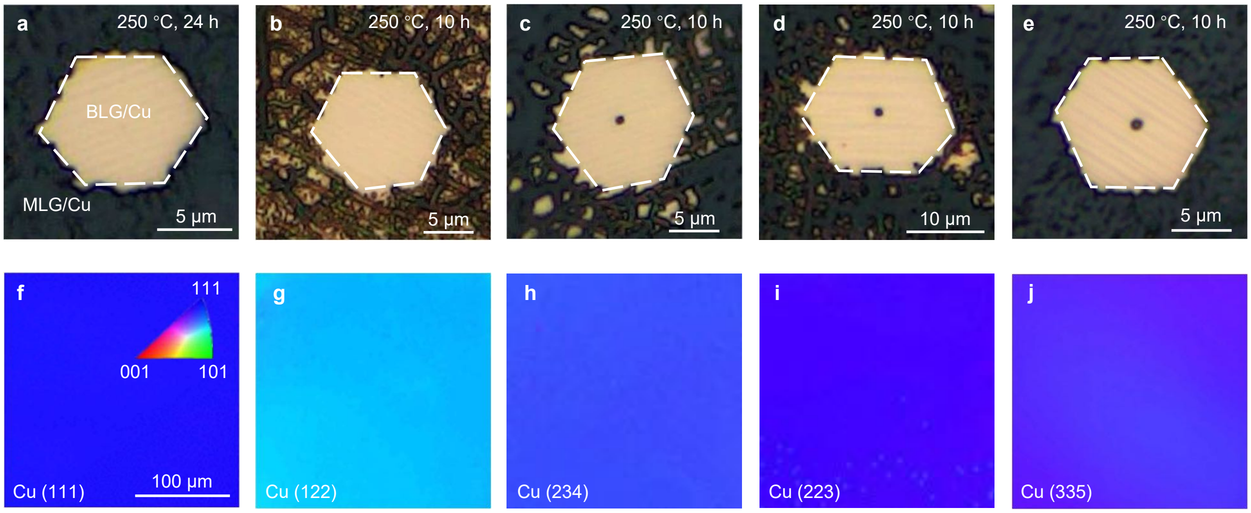

Supplementary Fig. 2 | Anticorrosion performance of bilayer graphene-coated Cu with different surface indices. a-e, Optical images of Cu with different surface indices after oxidation at 250 °C. **f-j**, Electron backscattered diffraction (EBSD) inverse pole figure (IPF) maps of the single-crystal Cu foils corresponding to (a-e). These maps have the same size.

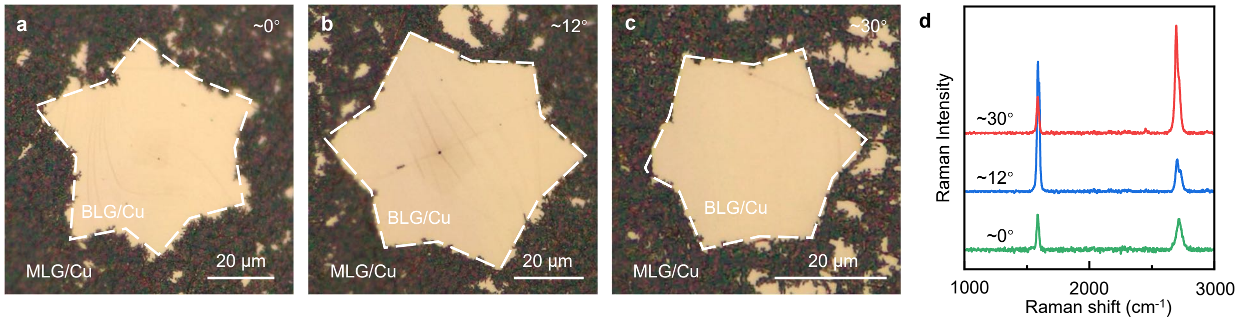

Supplementary Fig. 3 | Anticorrosion performance of bilayer graphene coated Cu with different twist angles. a-c, Optical images of twisted bilayer graphene coated Cu (0° (a), 12° (b), and 30° (c)) after oxidation at 250 °C for 10 hours. **d**, Raman spectra of the twisted bilayer graphene in (a-c), respectively.

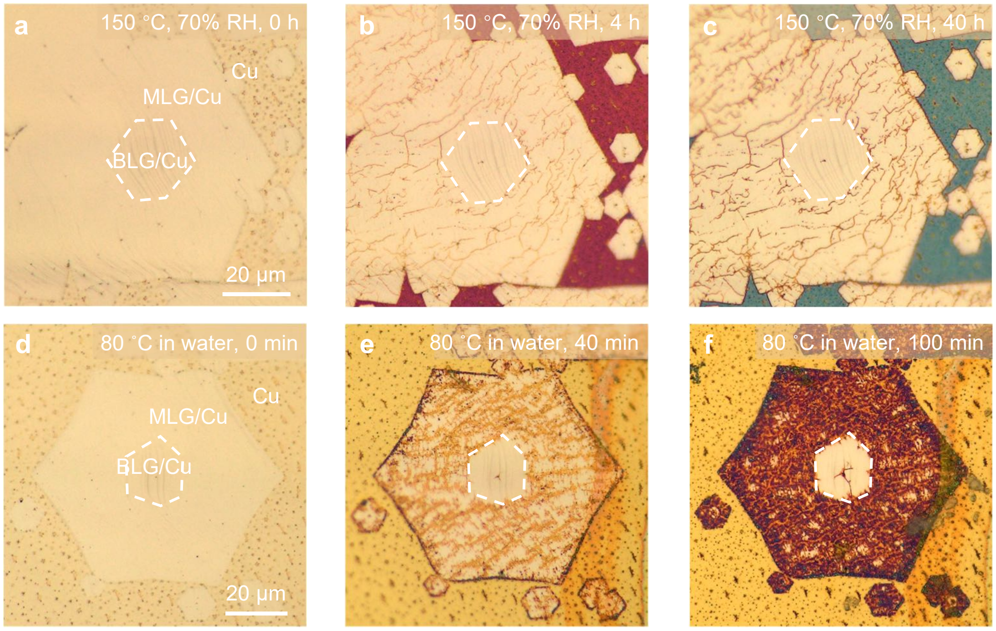

Supplementary Fig. 4 | Wet-oxidation of Cu with monolayer and bilayer graphene coating. a-c, Time-evolution optical images of monolayer and bilayer graphene coated Cu oxidized at 150 °C and 70% relative humidity (the relative humidity is measured at 25 °C). **d-f**, Time-evolution optical images of monolayer and bilayer graphene coated Cu oxidized at 80 °C in water.

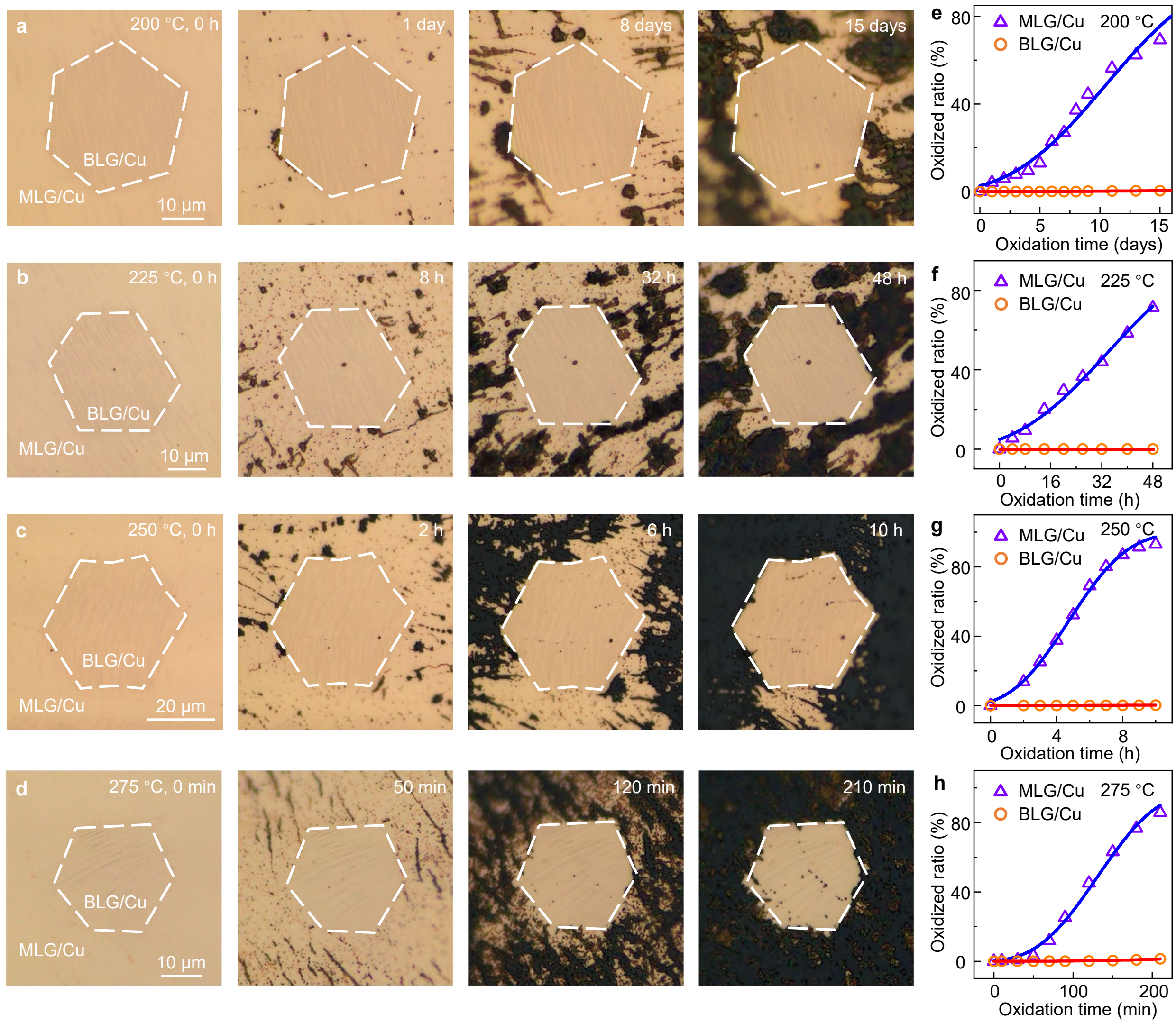

Supplementary Fig. 5 | Tracking of the oxidation process at different temperatures. a-d, Representative time-evolution optical images of the Cu surface oxidized at 200 °C, 225 °C, 250 °C, and 275 °C, respectively. **e-h**, Oxidized ratio at different temperatures of the monolayer graphene (triangles) and bilayer graphene (circles) coated area. The solid lines represent the fitting curves based on the two-step defect formation and expansion model.

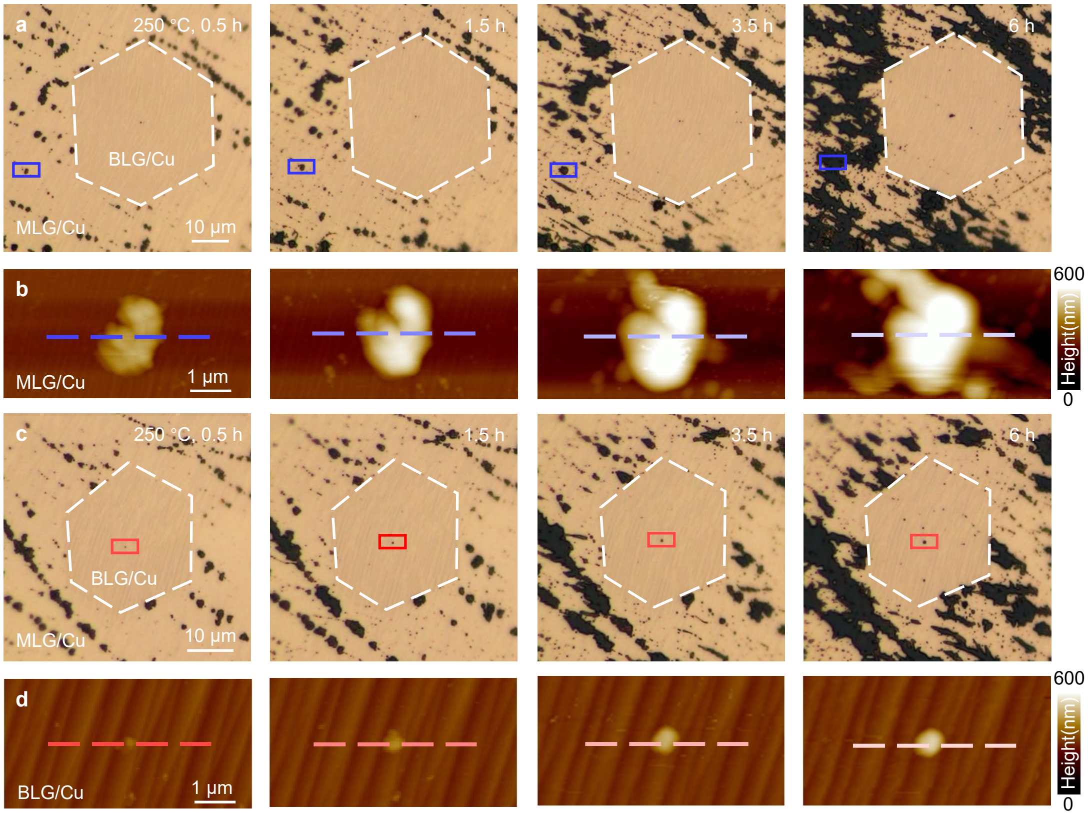

Supplementary Fig. 6 | Evolution of defects in the oxidation process. a, **c**, Time-evolution optical images of the defects on the monolayer and bilayer graphene coated Cu. **b**, **d**, AFM images of the defects as marked in blue (a) or red (c) solid rectangles, respectively. The corresponding time-evolution height profiles of these defects are summarized in Fig. 2f.

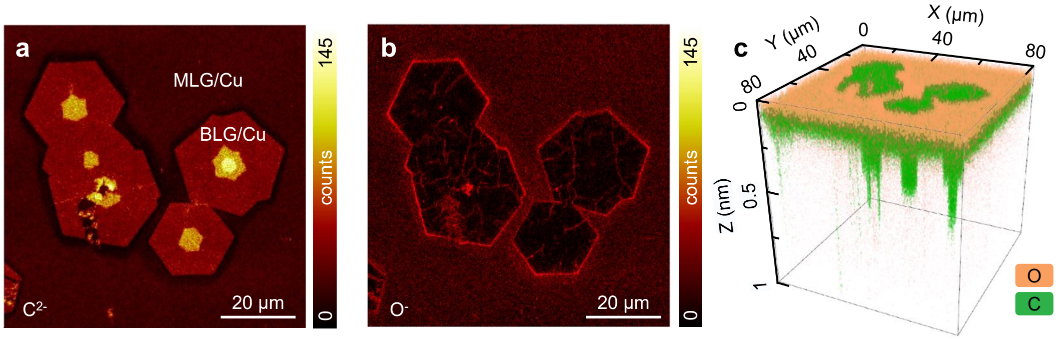

Supplementary Fig. 7 | ToF-SIMS characterization of graphene coated Cu after storage in air for 1 year. a, **b**, Element-distribution ToF-SIMS maps of C^{2-} (a) and O^{-} (b), respectively. **c**, Three-dimensional ToF-SIMS plot of O^{2-} and C^{+} distribution on monolayer and bilayer graphene coated Cu after oxidation. The vertical Z axis corresponds to the sample depth according to the irradiation time of the Cs^{+} clusters beam.

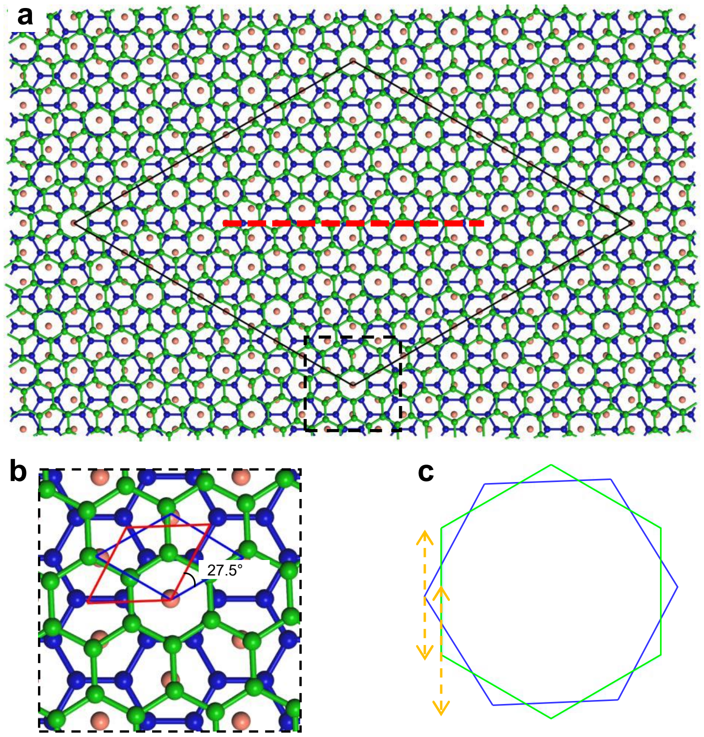

Supplementary Fig. 8 | The structure of Cu with bilayer graphene coating. a, The structure of Cu surface with bilayer graphene coating. The black diamond shape presents the lattice structure. The red dashed line indicates the cutting direction of electron density difference in Fig. 4a. **b**, Zoom-in structure of the graphene primitive cell in (a). The top and bottom layer has a 27.5° rotation. **c**, The Brillouin zones of the graphene layers. The green (blue) hexagon presents the first Brillouin zone of the top (bottom) layer graphene. The yellow arrows represent the positions of the ARPES results.

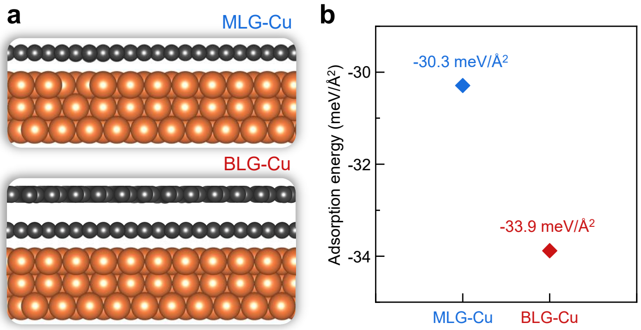

Supplementary Fig. 9 | The adsorption energies of monolayer and bilayer graphene on Cu. a, Schematic of the calculated final state structures of monolayer and bilayer graphene adsorption. **b**, The adsorption energies of monolayer and bilayer graphene on Cu.

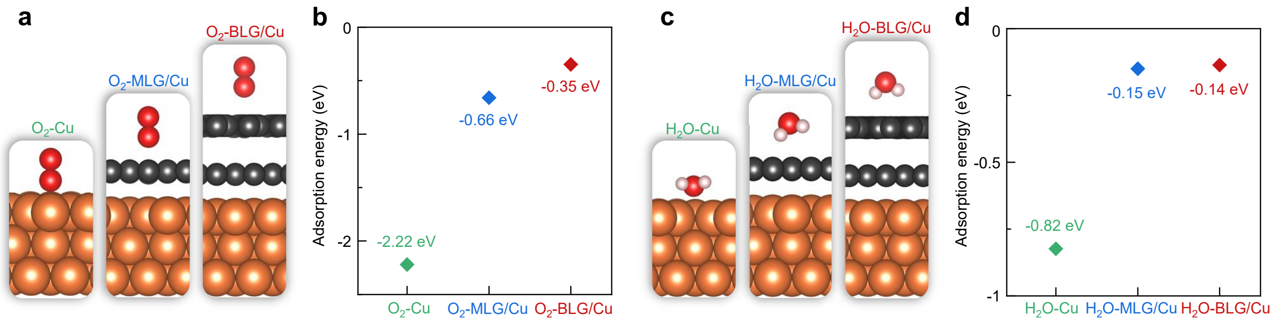

Supplementary Fig. 10 | The adsorption energies of corrosive molecules on bare Cu and graphene/Cu systems. **a**, **c**, Schematic of the calculated structures of oxygen (a) and water (c) adsorption. **b**, **d**, The adsorption energies of oxygen (b) and water (d) on the bare Cu, monolayer graphene on Cu, and bilayer graphene on Cu.

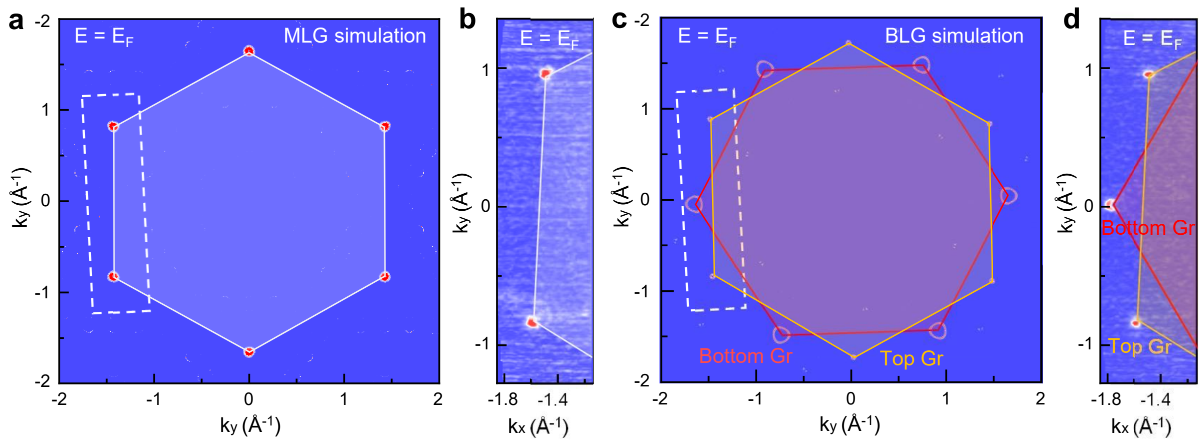

Supplementary Fig. 11 | Brillouin zone characterizations of monolayer and bilayer graphene on Cu. a, **b**, Calculated (a) and experimental (b) constant energy contours at the Fermi level of monolayer graphene. **c**, **d**, Calculated (c) and experimental (d) constant energy contours at the Fermi level of bilayer graphene. The first Brillouin zones of graphene are identified by the shadowed regions.

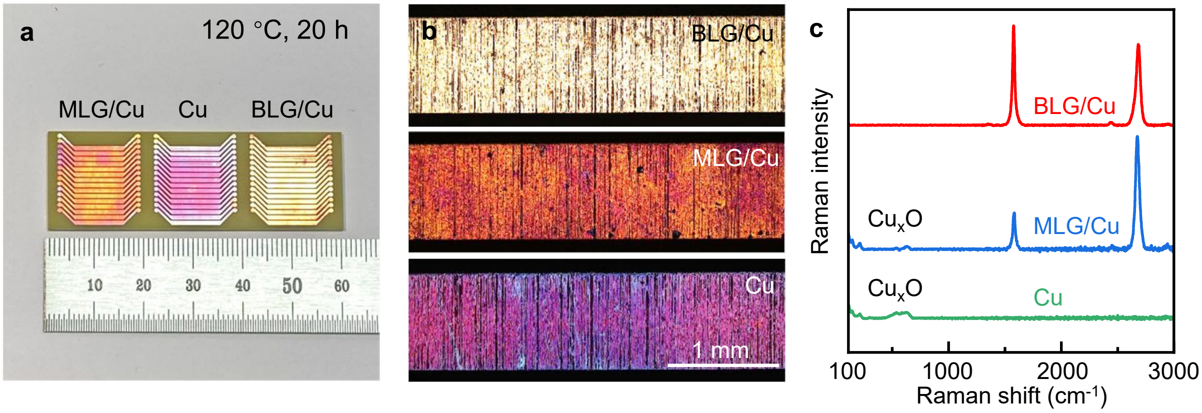

Supplementary Fig. 12 | Oxidation of graphene coated Cu conductors on a printed circuit board. **a**, Photograph of Cu conductors on a printed circuit board with monolayer coating, bilayer coating and bare surface after oxidation at 120 °C for 20 hours. **b**, **c**, Optical images (b) and Raman spectra (c) of the Cu conductors after oxidation.

Supplementary Table 1 | The fitted oxidation rates and oxidation limits for monolayer and bilayer graphene coated Cu.

Oxidation temperature		200 °C	225 °C	250 °C	275 °C
Oxidation rate (h^{-3})	MLG/Cu	1.2×10^{-8}	3.3×10^{-6}	1.9×10^{-3}	3.9×10^{-2}
	BLG/Cu	4.5×10^{-14}	1.1×10^{-10}	1.0×10^{-6}	3.0×10^{-4}
Oxidation limit (h)	MLG/Cu	~36	~3.6	<1	<1
	BLG/Cu	~1,100	~770	~37	~5.5
