# Supplementary material for: Enhanced copper anticorrosion from Janus-doped bilayer graphene
Source: Nat Commun. 2023 Nov 17;14:7447. doi: 10.1038/s41467-023-43357-1 (PMC10656578; doi:10.1038/s41467-023-43357-1)
Supplement: Supplementary file 3 — Description of Additional Supplementary Files [file 41467_2023_43357_MOESM3_ESM.pdf]

Description of Additional Supplementary Files

File Name: Supplementary Movie 1
Description: *In situ* SEM observation movie of the Cu oxidation process with graphene coating under 150 Pa O_2 and 80 Pa H_2 at 600 °C.
